# Evaluation of Microwave Vacuum Drying as an Alternative to Freeze-Drying of Biologics and Vaccines: the Power of Simple Modeling to Identify a Mechanism for Faster Drying Times Achieved with Microwave

**DOI:** 10.1208/s12249-020-01912-9

**Published:** 2021-01-19

**Authors:** Akhilesh Bhambhani, Justin Stanbro, Daniel Roth, Elizabeth Sullivan, Morrisa Jones, Robert Evans, Jeffrey Blue

**Affiliations:** 1 Vaccine Drug Product Development (VDPD), MRL, Merck & Co., Inc., West Point, Pennsylvania USA; 2 Process Development, Pilot Operations, Moderna, 200 Technology Square, Cambridge, Massachusetts 02139 USA; 3 Drug Product Development Operations, Teva Pharmaceuticals, 145 Brandywine Pkwy, West Chester, Pennsylvania 19380 USA; 4 Present Address: R.K. Evans Consulting LLC, 40 Mill St., Bangor, Maine 04401 USA

**Keywords:** biopharmaceuticals, lyophilization, microwave vacuum drying, semi-continuous, manufacturing, modeling, stabilization

## Abstract

Vial-based lyophilization for biopharmaceuticals has been an indispensable cornerstone process for over 50 years. However, the process is not without significant challenges. Capital costs to realize a lyophilized drug product facility, for example, are very high. Similarly, heat and mass transfer limitations inherent in lyophilization result in drying cycle on the order of several days while putting practical constraints on available formulation space, such as solute mass percentage or fill volume in a vial. Through collaboration with an external partner, we are exploring microwave vacuum drying (MVD) as a faster drying process to vial lyophilization wherein the heat transfer process occurs by microwave radiation instead of pure conduction from the vial. Drying using this radiative process demonstrates greater than 80% reduction in drying time over traditional freeze-drying times while maintaining product activity and stability. Such reduction in freeze-drying process times from days to several hours is a welcome change as it enables flexible manufacturing by being able to better react to changes either in terms of product volume for on-demand manufacturing scenarios or facilities for production (*e.g*., scale-out over scale-up). Additionally, by utilizing first-principle modeling coupled with experimental verification, a mechanism for faster drying times associated with MVD is proposed in this article. This research, to the best of our knowledge, forms the very first report of utilizing microwave vacuum drying for vaccines while utilizing the power of simplified models to understand drying principles associated with MVD.

## INTRODUCTION

Biopharmaceuticals, by virtue of their intrinsic instability, often rely on lyophilization as the current standard for product stabilization. In the last 30 years, the art of freeze-drying has advanced substantially and has been utilized extensively to overcome the instability barrier especially for aqueous biologics and vaccines. Substantial improvements, for example, in the sensor technologies and automation, enabled freeze-drying to be used as the current gold standard for drying biopharmaceuticals (1–4). Generally, the process includes placing aqueous or frozen vials of product on a temperature-controlled shelf, freezing the product and increasing temperature under vacuum conditions to drive sublimation and yield a dehydrated, stable product while keeping the product below its collapse (Tc) and/or glass transition temperature (Tg′) during the drying process. The potential for enabling room temperature stable products with improved patient compliance and simpler worldwide distribution remains a key business imperative for lyophilization of biopharmaceuticals.

Despite the benefits and advances, the process of freeze-drying remains an energy- and capital-intensive unit operation with limitations that prohibit its optimization for flexible manufacturing. Heat and mass transfer limitations inherent to lyophilization, for example, result in long drying cycles on the order of several days. Drug product formulations are typically constrained to excipients levels (*e.g.*, sugars, polyols) of usually ≤ 10% for improved stability (5). Increasing the solid content levels is anticipated to increase the mass transfer resistance and in turn may have corresponding negative consequences on product cost and production capacity. Choices for drug product primary containers are often limited too as a result of the heat and mass transfer limitations, and the extreme pressure and temperature conditions employed during the container sterilization and lyophilization process. The use of glass vials is nearly universal as alternatives, such as glass cartridges and recently emerging non-glass vials, have significant cost premiums and other technical challenges associated with them. The combination of multi-day drying cycles, scale-up challenges (especially with multiple units), and turnaround time for cleaning and sterilization can result in lyophilization process being the critical supply bottleneck, especially for products with variable market demand, short order lead times, and/or high volume needs to respond to outbreaks.

Now in the twenty-first century, various companies are actively pursuing alternatives to traditional freeze-drying in a vial so that processes can be modular, flexible, and “scaled-out” instead of “scaled-up” to meet diverse patient populations and different modalities, including small molecule, peptide, therapeutic protein, or vaccine (6). Unit operations of the future that rely on different heat transfer mechanisms to reduce cycle time (microwave drying, infrared drying, vacuum drying, spray drying) (7–10) or drying outside of the vial (spray drying, bulk drying, filter drying, *etc*.) (11–14) should enable flexibility that traditional batch freeze dryers cannot provide. It should be noted that many of these alternative drying approaches are uniquely utilized in the food industry in a reliable manner for commercial production of food in a non-GMP environment. However, their applicability for drying of pharmaceutical biologic materials at a large scale, while also complying with current Good Manufacturing Practice (cGMP) regulations, remains unknown. The purpose of this research is to explore microwave vacuum drying (MVD) technology as a rapid dehydration platform for freeze-drying of biologics and vaccines and utilize first-principle models to identify the mechanism for faster drying. A variety of different terms can be found in the literature for dehydration processes that incorporate microwave energy. Naming conventions like microwave vacuum drying (MVD), microwave-assisted vacuum drying (MAVD), and microwave-assisted freeze drying (MAFD) are generally subjective and not standardized. For the purpose of this publication, our definition of microwave vacuum drying is the application of microwave energy under deep vacuum levels (≤ 500 mTorr) to frozen material in a process comparable to traditional vial lyophilization. Furthermore, a specific form of MVD from EnWave Corporation, known as Radiant Energy Vacuum (REV™), was evaluated which relies on the application of non-ionizing microwave radiation at 2.45 GHz under vacuum to achieve fast dehydration. While microwave energy has been utilized as a common source for heating food in both household and industrial settings, its utilization in the pharmaceutical industry is severely limited partly due to limited understanding of (1) the interaction between microwave and the pharmaceutical products (including excipients) as well as (2) the approach for tuning the interactions to achieve uniform sublimation under vacuum conditions.

Gitter *et al*. recently published (15,16) their evaluation of microwave-assisted freeze drying to IgG1 type 1 monoclonal antibodies. Though we do not have first-hand experience with their specific dryer, their reported process appears comparable to REV™ drying in that it operates under deep vacuum with frozen product at the same microwave frequency. They demonstrated a 77% reduction in drying time compared to traditional lyophilization, with elegant cakes, and comparable accelerated stability. However, they did note challenges with microwave-assisted freeze-drying process that include a more difficult to control process compared to traditional lyophilization, risks of cold plasma discharge and batch inhomogeneity, and the need for designs that comply to pharmaceutical requirements.

To similarly assess these concerns, through a collaboration with an external partner, we have evaluated this proprietary REV™ drying technology as a potential to replace lyophilization as the workhorse dehydration approach for sensitive vaccine, biologic, and small molecule drug product. Because of the time-consuming nature, complexity, and variability associated with live virus potency assays, we evaluated two model proteins, catalase and hemoglobin, whose drying behavior is well-documented in the literature (17,18) prior to our exploration with a live virus vaccine.

Findings from our exploration successfully illustrate the potential of utilizing MVD as a faster alternative to freeze-drying of proteins and vaccines. Utilizing a lab-scale prototype, MVD was compared with traditional freeze-drying approach. In contrast to multi-day drying in lyophilization where cycles are frequently 48–72 h or longer, this research illustrates that rapid drying can be accomplished in MVD in approximately 6–12 h, representing an 80–90% reduction in cycle time, while maintaining product quality attributes and uniformity. Specifically, under the given experimental conditions, we were able to demonstrate > 80% reduction in drying time while maintaining product activity and stability. Furthermore, by utilizing first-principle models coupled with experimental verification, a hypothesis defining the mechanism for faster drying times achieved with microwave is also shared here. This research, to the best of our knowledge, forms the very first report of utilizing microwave vacuum drying for pharmaceutical drying of vaccines especially live virus vaccines (LVVs).

## MATERIAL AND METHODS

### Materials

Potassium phosphate monobasic (CAS 7778-77-0, P285-500) and potassium phosphate dibasic anhydrous (CAS 7758-11-4, P290-500) were obtained from Fisher Scientific. Sucrose (CAS 57-50-1, 7723-24) was obtained from Macron Fine Chemicals. Trehalose dihydrate (CAS 6138-23-4, BP2687-1) was obtained from Fisher Scientific. Hemoglobin from bovine blood (CAS 9008-02-0, H3760-100G), phenol red (CAS 34487-61-1) and catalase from bovine liver (CAS 9001-05-2) was obtained from Millipore Sigma. Tween™ 20 (CAS 9005-64-5) was obtained from Croda while 50% hydrogen peroxide (part number 21196-49) was obtained from Hach Company. All chemicals were research grade. Three cubic centimeter type I glass tubing vials with 13 mm opening (9621130307, 17 × 37.7 mm) were obtained from Gerresheimer while 13-mm igloo style lyophilization stoppers were obtained from West (10123723).

#### 0.5 mg/mL Hemoglobin, 5 mM Potassium Phosphate pH 7.2, 5% w/v Sucrose, 0.01% w/v Polysorbate-20

An 80% volume USP grade water was added to a beaker. Potassium phosphate monobasic and potassium phosphate dibasic anhydrous were added at a ratio to target pH 7.2. Sucrose and hemoglobin from bovine blood were added. The solution was mixed by using a stir bar, QS’ed with water, and sterile-filtered. PS-20 was added after sterile filtration.

#### 5 mg/mL Catalase, 50 mM Potassium Phosphate, pH 7.3

A total of 50 mg/mL of catalase enzyme was dialyzed using a 10,000 MWCO cassette (Slide-a-Lyzer, Cat 66,830) into 50 mM potassium phosphate, pH 7.3. The concentration was confirmed by UV absorption at 276 nm and the extinction coefficient from Millipore Sigma (12.9). The solution was then diluted to 5 mg/mL catalase in 50 mM potassium phosphate, pH 7.3.

#### Phenol Red–Layered Vials of 5% w/v Sucrose

To make layered frozen solutions for cake visualization, 0.3 mL of 5% w/v sucrose was added to 3-cc glass vials. Vials were frozen for at least 1 h in a – 70 °C freezer. Then, on dry ice, an additional 0.1 mL of 5% w/v sucrose, 0.6% w/v phenol red was added to minimize splashing on the walls. This was then quickly frozen using liquid nitrogen blast freezing. Finally, on dry ice, an additional 0.3 mL of 5% w/v sucrose was added and the vials returned to − 70 °C freezer overnight.

#### Vaccine Formulation

A live virus vaccine was formulated at 8% solids content and filled at 0.7 mL in 3-cc glass vials. Vials were frozen using liquid nitrogen blast freezing and then stored at − 70° C until drying.

### Microwave Vacuum Dryer and Lyophilizer

A lab-scale prototype freezeREV® dryer from EnWave Corporation (Canada) was used for all microwave drying runs. The dryer consisted of a vacuum chamber where the product was dried, a microwave chamber to house the microwave generating magnetrons with the waveguides and a dry ice condenser for capturing sublimated water vapor. Magnetrons for generating microwave were located below the drying chamber. A water load for capturing excess microwave was located above the drying chamber. Chamber pressure was maintained by running the vacuum pump continuously while bleeding room air into the chamber via a control valve set at 50 to 80 mTorr. The chamber shelf rotated the product around a center spindle at 1.25 RPM. The chamber can fit approximately 300 vials of ISO 2R size, depending on the packing arrangement. Because the chamber does not contain auto-stoppering, vials were manually stoppered after releasing vacuum. The microwave cycles presented in this publication demonstrate our increased understanding, optimization, and simplification of drying protocols.

All lyophilization studies were conducted with the lab-scale Lyostar® unit from SP Scientific (USA). The unit contained three shelves, which each could hold approximately 540 ISO 2R vials.

### Sublimation Mapping Using Gravimetric Analysis

**Fig. 1:**
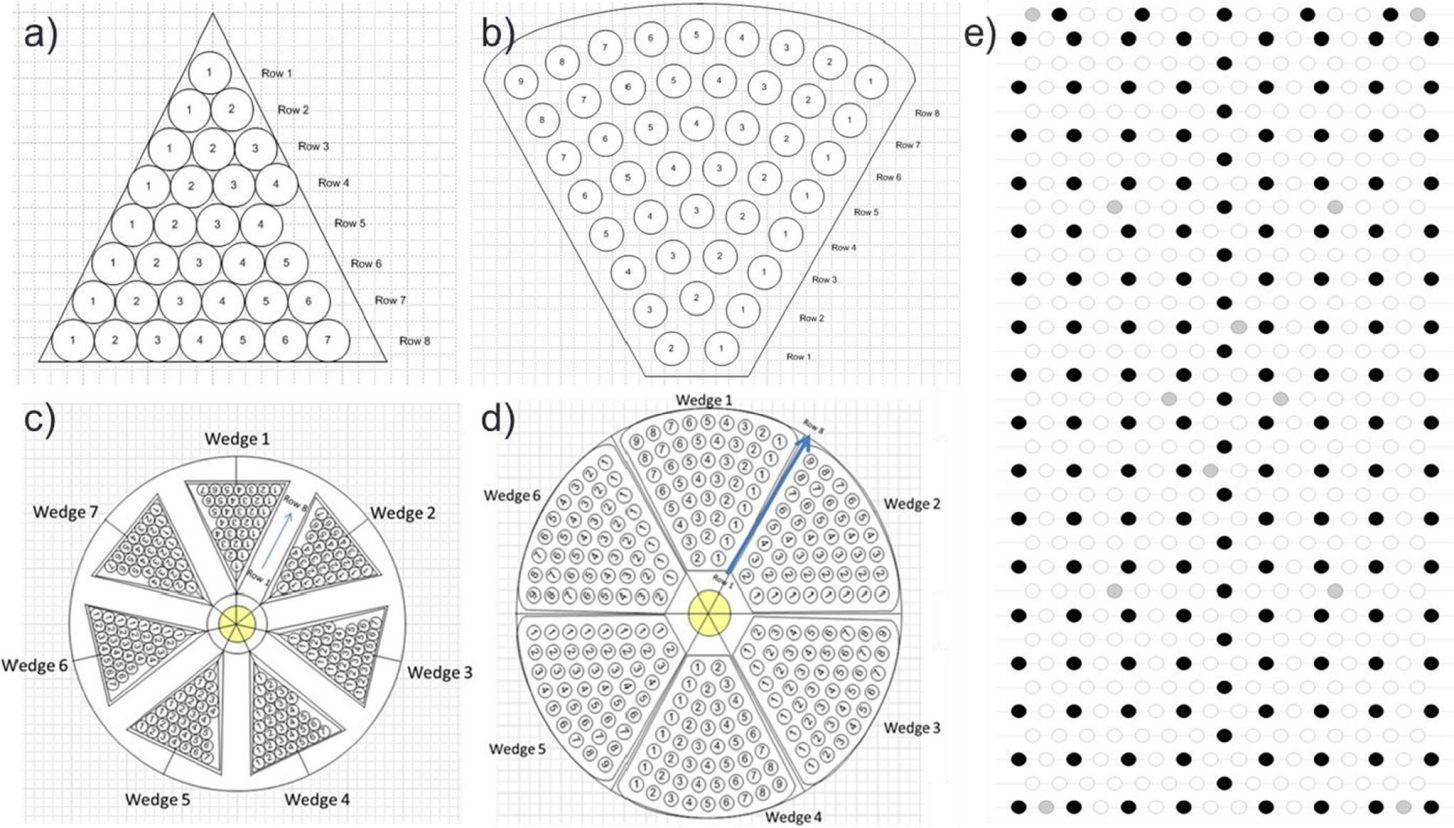
Vial packing arrangement for microwave dryer and lyophilizer trays. For microwave drying, vials were packed in either a tight (**a**) or loose (**b**) arrangement. For the tight arrangement, 7 wedges fit inside of the microwave chamber (**c**). For the loose arrangement, 6 wedges fit inside of the microwave chamber (**d**). For lyophilization, vials were packed in a traditional tight arrangement (**e**). Black circles represent active product. White circles represent placebo formulation. Gray circles represent placebo formulations with thermocouples

Three cubic centimeter vials were weighed empty on an analytical balance for the pre-use tare weight. Vials were then filled with 0.7 mL of solution and weighed again to obtain a pre-drying gross weight. Next, vials were packed into one of three arrangements as per Fig. 1. The microwave drying chamber contains a single circular shelf that rotates around a center spindle. Therefore, triangular PTFE trays were made to hold the vials in either a tight packing arrangement or loose packing arrangement. In the tight packing arrangement, 32 vials were placed inside of a triangular wedge so that adjacent vials were touching. Seven of these wedges were prepared per microwave run. In the loose packing arrangement, 44 vials were placed inside of triangular trays with holes that rigidly space the vials 1″ apart from each other (center to center). Six of these wedges were prepared per microwave run. For lyophilization, a single 11″ × 20″ bottomless, stainless steel tray was used which holds 543 vials. The tray consisted of both active hemoglobin vials and placebo vials as per Fig. 1. The placebo solution was identical to the hemoglobin solution except that it only contained potassium phosphate and sucrose. Select placebo vials had thin-wire thermocouples placed at the bottom center of the vial.

Prepared trays for drying were next flash frozen using liquid nitrogen *via* a custom blast freezer at – 115 °C for 15 min. Vial trays were stored at – 70 °C until drying.

Next, vial trays were transferred on dry ice to either the microwave dryer or the lyophilizer, where they were dried by protocols described below. Vials were not stoppered for all drying runs. After drying, vials were immediately stoppered, capped, and stored until post-drying weighing. When the vials had equilibrated to room temperature, the stoppers and caps were removed and the vials were weighed for a post-drying gross weight. The amount of water removed by sublimation was calculated by subtracting the post-drying net weight from the pre-drying net weight.

All microwave runs were based on a benchmark drying protocol (Table I) but stopped at varying cycle durations to create samples for “one-third,” “one-quarter,” “one-half,” and “full” cycles (Table II). For example, the “full” cycle was 6.2 h long and the “one-quarter” cycle was 1.5 h long. The microwave exposure settings during the 90 min for one-quarter of cycle were identical to the first 90 min of the “full” cycle. The microwave energy was supplied by 4 magnetrons which cycled on and off *via* a pre-defined algorithm to minimize the potential for hot and cold spots.

**Table I:** Microwave Parameters for “Full” Cycle

Exposure time (minutes)	Number of magnetrons	Power per magnetron (watts)	Total power (watts)	Vacuum setting (mTorr)
30	4	50	200	50–80
60	4	100	400	50–80
120	4	200	800	50–80
90	4	400	1600	50–80
60	4	200	800	50–80

**Table II:** Definition of Partial Cycles

Cycle name	Total run time (hours)	Total energy exposure (kWh)
Quarter	1.5	0.5
Third	2.25	1.0
Half	3.5	2.0
Full	6.2	5.1

**Fig. 2:**
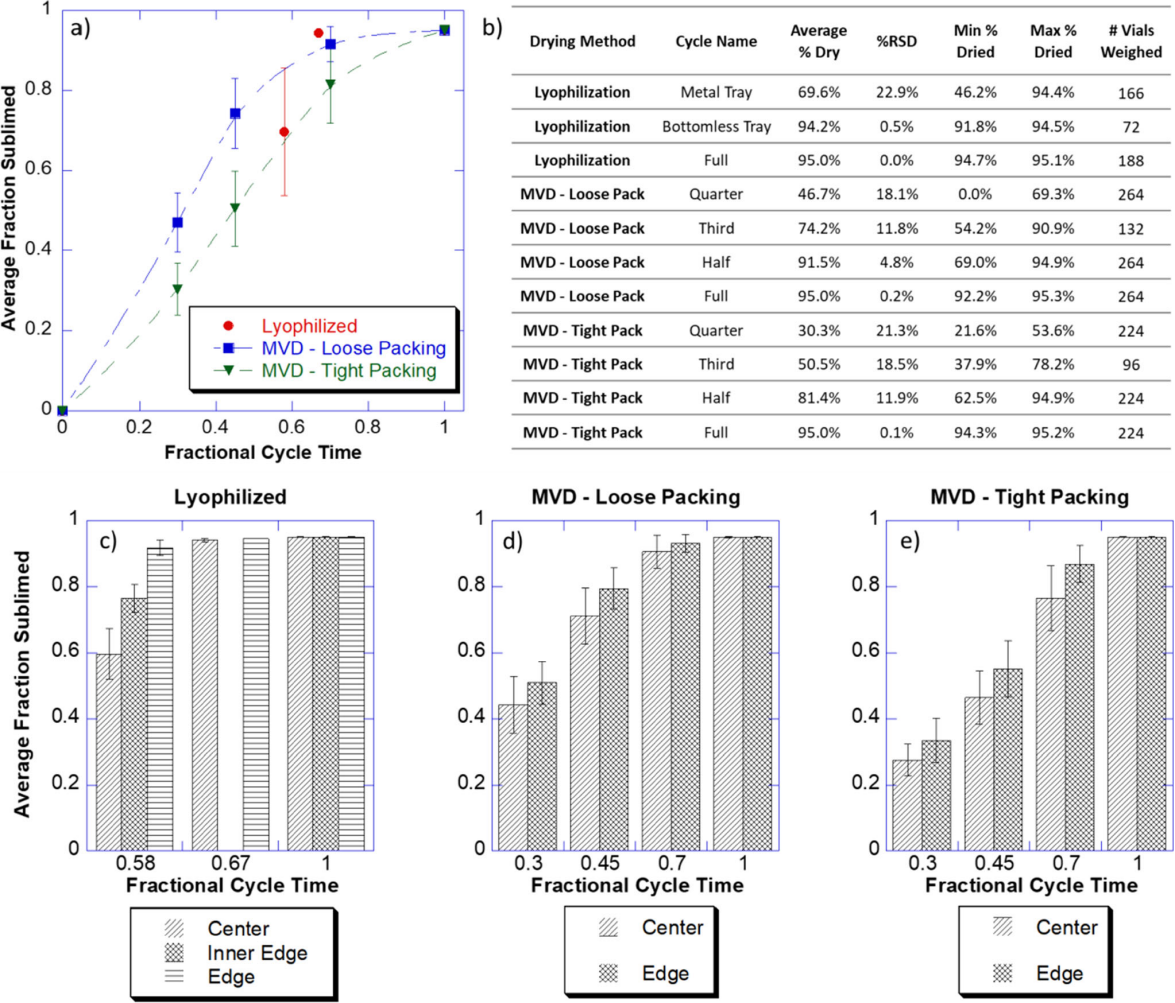
Sublimation study results. **a** Average fraction sublimed *vs*. fractional cycle time. The *x* axis represents a normalized cycle time so that the MVD and lyophilization cycles can be compared. The MVD cycle was 6.5 h long whereas the lyo cycle was approximately 50 h long. Each data point represents the average from a single drying run. Error bars represent one standard deviation of all weighed vials, as listed in inset **b**. **b** Statistical summary of the gravimetric sublimation data from each run. Graphs **c**, **d**, and **e** present the same data, but grouped by location within the tray. Edge vials are considered the outermost perimeter vials in a tray. For MVD, center vials are any other vial in that tray. Because the lyophilization tray is much larger than an MVD tray, a third group called inner edge is defined as vials that are not edge vials but are not surrounded by 6 vials because they are one vial in from the edge. Error bars represent one standard deviation. Note that for the 0.67 fractional lyophilization cycle, inner edge vials were not weighed because there was no substantial difference between center and edge vials

Three lyophilization cycles were completed using permutations of the cycle shown in Table III for comparison to the microwave dryer sublimation data. All 3 cycles were conducted using the top shelf of a Lyostar 3 with a metal door. In the first run, the cycle in Table III was run to completion using a bottomless tray. In the second and third runs, the step 2 hold time was reduced to 735 min and no secondary drying was performed. Run 2 was conducted with a bottomless tray whereas run 3 was conducted with a metal-bottomed tray, to vary sublimation rates. Findings from MVD runs were compared against lyophilized vials (Fig. 2).

**Table III:** Lyophilization Cycle

Parameter	Primary		Secondary
Step	1	2	3
Temperature (°C)	− 50	− 15	30
Ramp rate (°C/min)	0	1	0.05
Hold time (min)	30	1645	300
Vacuum (mTorr)	40	40	40

### Cake Visualization Using Vial with Dye

Twenty frozen vials of the phenol red–layered solution were microwave-dried using the cycle in Table IV. In parallel, 10 frozen vials were lyophilized using the cycle in Table III. After drying, vials were stoppered and stored at room temperature.

**Table IV:** Microwave Drying Cycle for Cake Visualization Study

Exposure time (minutes)	Number of magnetrons	Power per magnetron (watts)	Total power (watts)	Vacuum setting (mTorr)
60	1	400	400	50–80
120	2	400	800	50–80
60	3	400	1200	50–80
60	2	400	800	50–80

**Fig. 3:**
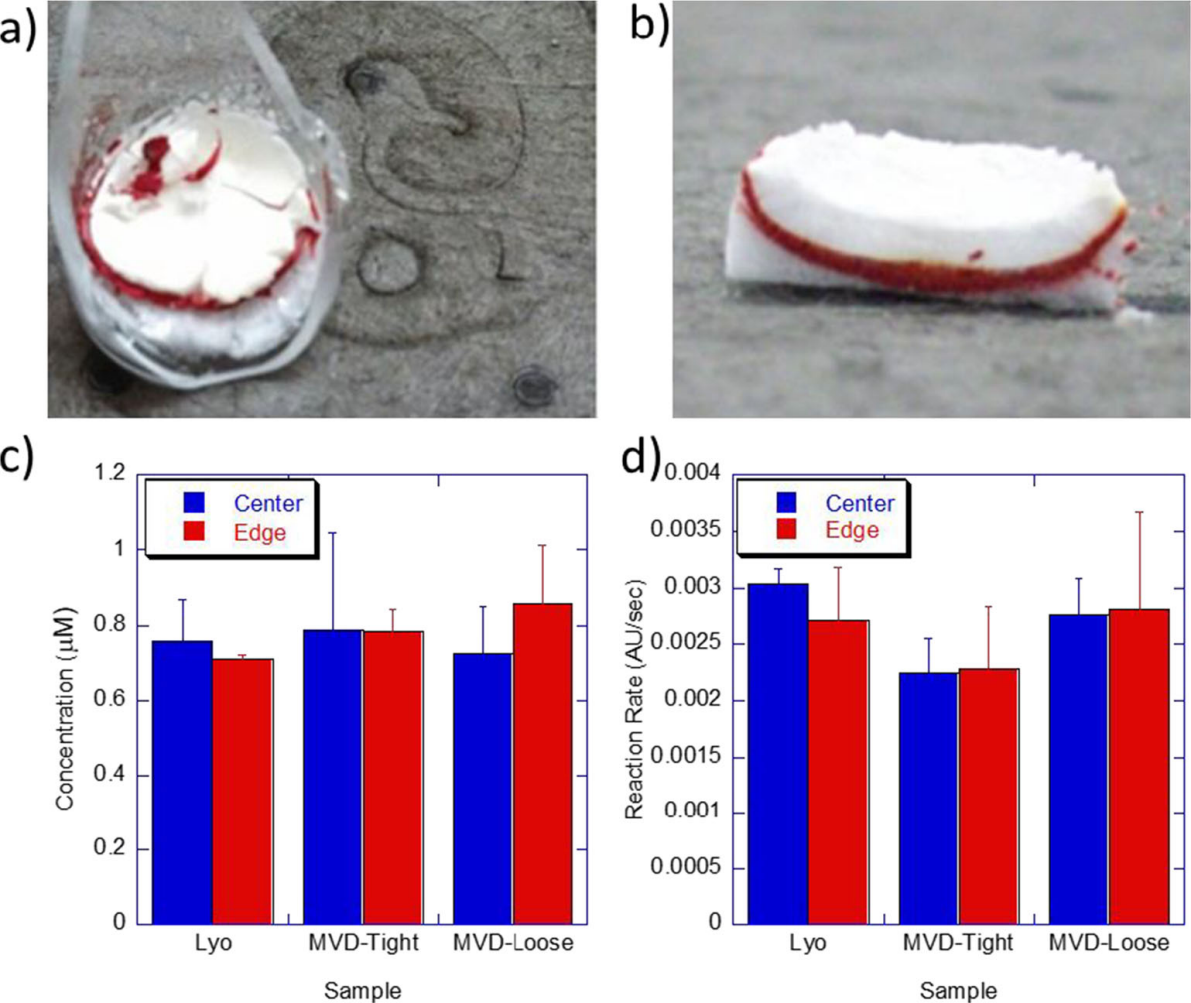
Cake visualization and activity retention. Dissected microwave-dried cake (**a**) and lyophilized cake (**b**) showing no signs of dye migration. **c** Concentration of hemoglobin in the center *vs.* edge of dissected cakes dried by lyophilization, MVD in a tight packing arrangement, and MVD in a loose packing arrangement. **d** Rate of reaction of guaiacol plus hemoglobin from the center *vs.* edge of dissected cakes dried by lyophilization, MVD in a tight packing arrangement, and MVD in a loose packing arrangement. Reaction rate was determined by measurement at 470 nm after addition of sample, guaiacol, and H_2_O_2_. Error bars represent one standard deviation of the mean for 3 samples

For visual analysis, vials were scored using a glass cutter and then cracked open. Any broken glass pieces were removed. The cake was then cross-sectioned using a scalpel (Fig. 3).

### Drying Evaluation Using Model Proteins

Catalase was used as a model protein to study the drying impact. ISO2R vials were filled at 0.7 mL and frozen using liquid nitrogen blast freezing. Nineteen vials were dried in the microwave vacuum dryer using the cycle in Table V. Nineteen separate vials were dried in the lyophilizer using the cycle in Table VI, and catalase drying cycle parameters were adapted from Lale *et al*. (17). Dried vials were stoppered and stored at – 70 ^o^C until activity determination.

**Table V:** Microwave Drying Cycle for Model Protein Activity Study

Exposure time (minutes)	Number of magnetrons	Power per magnetron (watts)	Total power (watts)	Vacuum setting (mTorr)
240	2	200	400	50–60
240	3	200	600	50–60

**Table VI:** Lyophilization Cycle for Protein Activity Study

Step	1	2	3	4	5	6	7
Temperature (°C)	− 40	− 35	0	10	15	25	30
Ramp rate (°C/min)	1	1	1	1	1	1	1
Hold time (min)	20	1000	30	10	10	10	420
Vacuum (mTorr)	500	500	200	200	200	200	200

A follow-up microwave drying study was performed where the terminal temperature was monitored using a thermal imaging camera. In this study, 3 separate trays of 19 vials were dried in the microwave vacuum dryer using the cycle in Table VII. The cycle was paused at 270 min and the first tray of vials removed. The cycle resumed for another 55 min at a higher power before the cycle was paused and the second tray removed. Finally, the cycle was allowed to complete when the last tray was removed. All vials were stoppered and stored at − 70°C until activity determination.

**Table VII:** Microwave Drying Cycles for Varying Terminal Temperature

Exposure time (minutes)	Number of magnetrons	Power per magnetron (watts)	Total power (watts)	Vacuum setting (mTorr)
190	2	200	400	50–60
80	2	300	600	50–60
55	2	450	900	50–60
65	3	400	1200	50–60

#### Catalase Activity

Frozen control samples were prepared by thawing at room temperature. Dried samples were prepared by reconstituting with 0.7 mL water. Once samples were in the liquid state, they were serially diluted to 0.05 mg/mL using 50 mM potassium phosphate, pH 7.3. The concentration was confirmed using UV absorption at 276 nm and the extinction coefficient from Millipore Sigma (12.9 L/mol/cm). A total of 2.9 mL of 0.036% hydrogen peroxide was added to a 3-mL quartz cuvette with 1-cm path length. The cuvette was read using a kinetic scan on an Agilent UV/Vis spectrophotometer for 120 s, sampling every 1 s at 240 nm. At 20 s, 100 μL of the diluted catalase sample was added to the cuvette and pipetted up and down 5 times to mix. The data was analyzed using an extinction coefficient of 35 L/mol/cm for hydrogen peroxide at 240 nm. The enzymatic activity was calculated using the slope following catalase addition.

#### Hemoglobin Peroxidase Activity

Lyophilized and microwave-dried hemoglobin samples from above were dissected so that the center and edge of the cake could be tested for peroxidase activity as described in literature (19). Material from the center or edge was carefully removed with a metal scoop and weighed into new vials for analysis. Samples were then reconstituted with water to target the same protein concentration as prior to drying. Protein concentration was determined by measuring absorbance at 410 nm on an Agilent UV/Vis spectrophotometer with 303,956 cm^−1^ M^−1^ extinction coefficient. Peroxidase activity was determined by measuring the absorbance change at 470 nm for 2 min, following the addition of 25 μL sample, 175 μL buffer, and 25 μL H_2_O_2_.

### Microwave Absorption Study

Solutions of 5%, 10%, 15%, and 20% w/v sucrose were prepared and filled into 3-cc vials at 1 mL fill volume. Vials were weighed and then frozen using liquid nitrogen blast freezing. Per formulation, 19-vials were dried in constant power microwave cycles for 60 min at 50–60 mTorr. Separate vials were used for each of the microwave cycles at 0, 540, 1080, and 1600 W. Vials were weighed after the cycle to determine the amount of water removed per hour.

#### Specific Surface Area Measurements

**Fig. 4:**
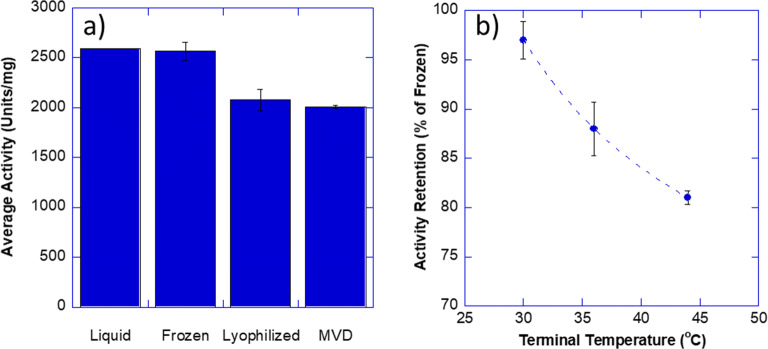
Catalase activity following lyophilization and MVD. **a** Activity of 5 mg/mL catalase for liquid, freeze/thaw, lyophilized, and MVD samples. **b** Activity of microwave-dried catalase as a function of terminal temperature

A total of 5% w/v sucrose solution was filled into 3-cc vials at 0.7 mL fill volume and then frozen using liquid nitrogen blast freezing. A total of 152 vials were dried in the microwave vacuum dryer using the cycle in Table IV, but with vacuum set to 50–60 mTorr. A total of 227 vials were dried in the lyophilization cycle in Table III, except that the primary drying temperature was set at − 21°C instead of − 15°C. Product temperature was determined using fiber optic thermocouples for the microwave dryer samples and thin-wire metal thermocouples in the lyophilized samples. Thermocouples were placed into the bottom center of the respective vials before freezing. After drying, all samples were stoppered and stored at − 70°C until analysis (Fig. 4).

#### Specific Surface Area Determination

Specific surface area was conducted by Micromeritics Pharmaceutical Services (Norcross, GA). The masses of eight 3/8″ TriStar tubes were recorded and then placed inside a glove box. Each sample was prepared by breaking up the cakes from several vials per sample type. Enough of each sample was used to fill the bulb of the TriStar tube. Once all tubes were filled, the masses of the tube plus sample were recorded. The samples were then placed on the ASAP 2420 to degas under vacuum at 25 °C for 960 min. Following the degas procedure, the tube masses were recorded for a final sample mass. A filler rod was placed in each of the tubes and an isothermal jacket around it. The tubes were attached to the Tri Star to begin the automated analysis procedure.

#### MicroCT

Dry cake pictures were imaged *via* Xradia VersaXRM-500 using the following settings. To increase contrast, samples marked for imaging were spiked with Omnipaque solution.

- Source voltage: 80 kV
- Source power: 7 W
- Source distance: − 50 mm from the sample
- Detector distance: 130 mm from the sample
- Objective: × 0.39
- Exposure time: 1
- Start angle: − 180
- End angle: 180
- # of images: 720

### First-Principle Modeling

First-principle equations for steady-state heat and mass transfer during lyophilization are well-known in the literature (20,21). These equations are used to determine how sublimation rate during primary drying can be optimized by modulation of the vial heat transfer coefficient and the formulation mass transfer resistance term. For the purposes of this research, these equations were used to qualitatively compare the differences between heat and mass transfer attributes in MVD to lyophilization. Where calculations were performed, they were done in Microsoft Excel.

### Moisture Analysis

Moisture analysis was carried out using the 831 KF coulometer, a Karl Fischer system from Metrohm. Samples were reconstituted using 3 mL of 50% v/v formamide and 50% v/v methanol. A total of 1 mL of the 3 mL reconstituted solution was injected into the reagent reservoir containing Hydranal-Coulomat AG. The amount of water in the reconstitution solution was subtracted from the sample results. The resulting water content was divided by the net weight of the sample to determine the residual moisture content.

### Drying Evaluation of Live Virus Vaccine

Frozen live virus vaccine was dried in the microwave vacuum dryer using the cycle in Table VIII or in the lyophilizer using the cycle in Table III, except that the hold time for step 2 was 3 h longer. Post drying, vials were stoppered, capped, and stored at accelerated stability conditions (2–8 °C). As time points were pulled, samples were stored at – 70 °C until the end of study. Relative potency was determined at time zero and then for all samples at the completion of the study.

**Table VIII:** Microwave Drying Cycle for SSA Sample Preparation

Exposure time (minutes)	Number of magnetrons	Power per magnetron (watts)	Total power (watts)	Vacuum setting (mTorr)
30	4	50	200	50–80
60	4	100	400	50–80
120	4	200	800	50–80
120	4	300	1200	50–80
60	4	200	800	50–80

#### Live Virus Relative Potency

Relative potency was determined using a 96-well format ELISA-based assay. Frozen samples were thawed on the benchtop. Dried samples were reconstituted with 0.7 mL water. Four vials per drying technology and time point were tested for replication. Relative potency was compared to a standard and the log potency loss was determined by comparison of each time point to the post-drying, time-zero sample.

#### Results

Microwave vacuum drying serves as a faster alternative to freeze drying of biologically active materials such as vaccines, proteins, and microorganism cultures. Our research explorations for biologics and vaccines drying were inspired by the fact that drying in food industry using MVD occur in hours and not days. It should be noted that despite widespread application of microwaves globally in households and various food industry, the application of MVD in pharmaceuticals is severely limited due to concern with depth of penetration, uncontrolled heating and lack of mechanistic understanding of drying process required to establish a uniform and repeatable processes that can be scaled-up.

A systematic approach to evaluate potential concerns of achieving uniform drying at relatively high sublimation rate was addressed using hemoglobin and catalase as surrogate proteins wherein the cake appearance, sublimation rates, and protein activities were characterized and compared against freeze-dried products. To better understand the mechanism of interaction between microwave radiation and excipients, a series of increasing sucrose formulations were dried in microwave vacuum dryer and their absorption efficiency was characterized. Furthermore, product temperature profile was obtained from a freeze dryer run and compared against the MVD. When complemented with SSA (specific surface area) analysis and modeled using first-principle heat and mass transfer mechanisms for freeze-drying phenomenon and radiative drying, we conclude that rapid drying is attributed to volumetric energy transfer through microwave radiation wherein energy is transferred to the product plug throughout its volume. Such heating is impacted by the dielectric and loss factor of the formulation without being constrained by the apparent heat coefficient of a vial (*K*_v_). This contrasts with freeze-drying wherein the heat transfer for sublimation occurs through conduction from the bottom of a vial and *K*_v_ is an intrinsic property of the vial and is not contingent upon excipients. Furthermore, whereas the presence of a higher solute concentration in the freeze-drying process generally results in an increase in the mass transfer resistance, for MVD, this may improve the interactions of microwave radiation through the dielectric and loss factor of the excipients.

### Sublimation Rate and Uniformity of Microwave Drying Compared to Lyophilization

The goal of our first study was to characterize the speed, uniformity, and repeatability of MVD compared to lyophilization. We used a model protein formulation (0.5 mg/mL hemoglobin, 5 mM potassium phosphate pH 7.2, 5% w/v sucrose, 0.01% w/v polysorbate-20) at 0.7 mL fill volume in 3-cc glass vials. After establishing a full MVD cycle (Table I), we repeated the cycle, but stopped it at specified times to define various partial cycles. Each cycle was conducted independently and consisted of gravimetric analysis before and after the run to determine the amount of water removed. These experiments were also performed using a lyophilization cycle. That cycle was then also repeated but stopped at various points during primary drying to define the partial cycles.

The MVD cycle was 87% shorter than the lyophilization cycle and drying was completed in 6.5 h in MVD (approx. 50 h in lyophilization). To compare the heterogeneity between microwave vacuum drying and lyophilization, we first defined the lyophilization cycle based on the amount of time it took to reach thermocouple convergence in the benchmark cycle (1120 min). Then, as an example, if the subsequent partial lyophilization cycle was stopped at 750 min, its fractional cycle time was documented as 0.67. It should be noted that cycles were conducted on a different style tray (rectangular tray for lyophilization and circular for MVD), and thus, an empirical factor was applied to account for the difference in heat transfer and the partial cycles were normalized relative to the full cycle time (370 min) minus the secondary drying hold time (60 min). Thus, as an example, if the partial microwave cycle was stopped at 210 min, its fractional cycle time was 0.68.

After normalizing the cycle times, we then compared the average fraction of water sublimed to the normalized fractional cycle time (Fig. 2). We observed that the microwave-dried vials follow a similar “path” as one might expect from a lyophilization process, however, in a much faster time scale. For example, approximately half-way through our cycle, in line with expectation, approximately half of the water was sublimed. There are of course locational differences within a lyophilization cabinet, which were evaluated next.

By tracing each vial’s weight to its location in the chamber, we could determine the overall sublimation heterogeneity at any point in the drying time. Comparing standard deviations (the error bar in Fig. 2a) and relative standard deviations (table inset in Fig. 2), we saw comparable variability when primary drying had not completed. Similar to lyophilization, edge vials dried faster than center vials in the microwave dryer (Fig. 2c, d, e). Additionally, there appeared to be greater variability between center and edge vials during the lyophilization cycle stopped at 58% complete (Fig. 2c) compared to the MVD cycles stopped at any point less than 100% complete (Fig. 2d and e). For example, when the lyophilization cycle was stopped at 58% complete, the center vials had an average of 59.6% of the water removed and the edge vials 91.8% of the water removed. In contrast, MVD in a tight pack arrangement stopped at 70% cycle completion demonstrated averages of water removed of 76.5% and 86.9% for center and edge vials, respectively. This observation may likely be dependent on tray size, which for lyophilization was much larger than for this prototype MVD.

Finally, because the sublimation data came from multiple independent drying cycles, we could evaluate some level of repeatability from the data. For each experiment, as we increase the amount of drying time, we see an increase in the amount of water removed in a consistent manner until we approach the end of drying.

### Cake Visualization and Protein Activity

We next wanted to evaluate the mechanisms involved with faster sublimation observed in microwave vacuum drying. Although we hypothesized that heat transfer was a key factor, we wanted to understand the impact of mass transport differences between microwave drying and lyophilization. To make progress towards this goal, we created layered frozen solutions of 5% w/v sucrose with and without phenol red. We then dried the cakes in either the microwave vacuum dryer or the lyophilizer. If macroscopic changes were taking place in the cake with gross phase changes from solid to liquid, color changes in the layers would be detected, such as a migration or diffusion of the red dye into the undyed portions. However, after dissecting both sets of samples (Fig. 3a and b), we observed no visual differences in the dye location between either set of dry samples.

To further assess if there were location-dependent interactions with microwave energy or gross changes within the cake, the lyophilized and microwave-dried hemoglobin samples from above were also dissected. The centers of the dried cakes were carefully removed so that both the center and edge could be characterized for protein concentration and hemoglobin peroxidase activity. It is well-reported in literature that oxidation of 4-methoxyphenol (guaiacol) occurs in the presence of hydrogen peroxide and hemoglobin, which results in the formation of colored tetrameric product with a spectroscopic signature at 470 nm (19). Thus, absorption at 470 nm was monitored for both lyophilized, and MVD samples and the first-order growth rates were nearly the same for both samples (Fig. 3d). Furthermore, the protein concentration within the center of the cake was comparable to that within the edge of the cake, when measured by absorption at 410 nm (Fig. 3c). These results demonstrate the absence of gross melting or phase change during the microwave vacuum drying process, accompanied with no loss of protein activity, even though the MVD process is a substantially faster dehydration approach.

To further understand if the microwave vacuum dryer was negatively impacting active products in general, we evaluated the activity retention of catalase enzyme as an alternate model protein. Enzymatic activity was determined by observing the catalytic reduction in the concentration of hydrogen peroxide as a function of time. The fresh liquid sample and the freeze/thaw sample had similar activities (Fig. 4a). As expected, the lyophilized product demonstrated some loss of activity with an 80.9% recovery of activity when compared to the freeze/thaw sample. The microwave-dried sample had a similar yield of 78.3% demonstrating comparability between MVD and freeze-dried samples. Furthermore, recognizing that activity retention could be impacted by the terminal temperatures reached in the drying cycles, we performed a follow-up study in the microwave dryer where terminal temperature was altered. Three trays of vials were dried at the same time. When the average product temperature as determined by a thermal imaging camera in the ceiling of the machine reached 30 °C, the cycle was paused, and the first tray was removed. The cycle continued until the average product temperature reached 36°C and then the second tray was removed. Finally, the last tray was dried until the average product temperature reached 44 °C. The activity of each tray was then determined as above and plotted as a function of terminal temperature (Fig. 4b). As temperature increased, the activity retention decreased from 97% at 30 °C to 81% at approx. 45 °C. The lyophilization activity retention, on the other hand, was 80.9% when the samples were dried to 30 °C terminal temperature (Table VI). The higher activity retention in MVD when dried to 30 °C was not expected and may be a result of either drying differences or the amount of time spent at elevated temperatures.

### Specific Surface Area

**Fig. 5:**
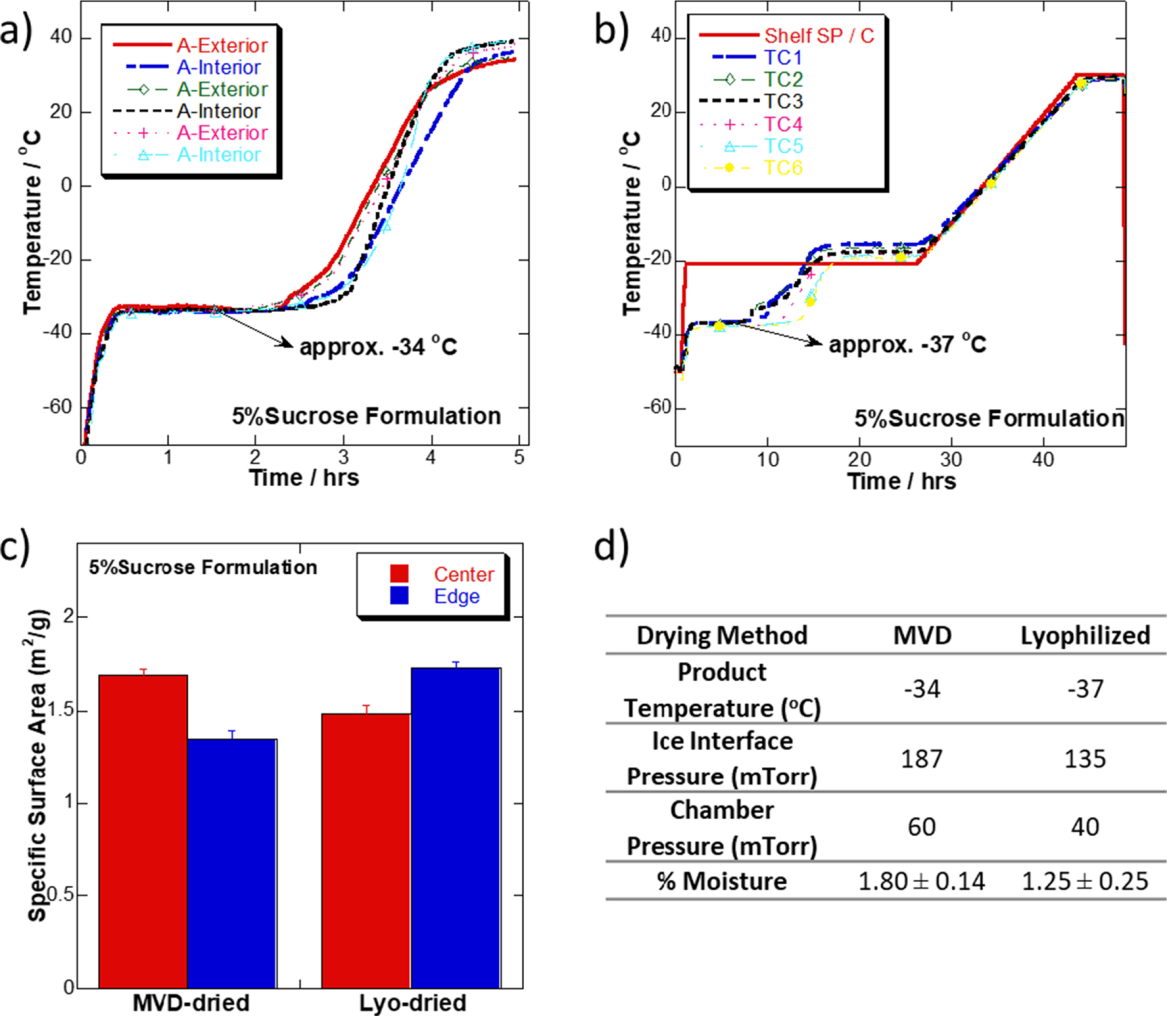
First-principle modeling of steady-state mass transfer resistance. Product thermocouple traces for a 5% w/v sucrose solution dried *via* **a** MVD and **b** lyophilization. **c** Specific surface area results for MVD and lyophilized samples. **d** Corresponding parameters for mass transfer comparison of MVD and lyophilized samples

After observing that microwave-dried cakes were dried much faster than lyophilization without macroscopic cake alteration or loss of enzymatic activity, we turned to the steady-state heat and mass transfer models to better understand the process. These models, used frequently in the literature to characterize the primary drying design space, allow us to predict how sublimation rate will change as product temperature and/or mass transfer resistance are changed (22). We speculated that MVD may be sublimating faster either because of increased heat transfer at higher product temperature or because of microscopic changes in the cake that lead to a decrease in the mass transfer resistance. Figure 5a and b show that the average product temperature (*T*_p_) in the microwave dryer (− 34 °C) was in fact warmer than that in the lyophilizer (− 37 °C). This 3-degree increase in product temperature increases the ice interface pressure from 135 to 187 mTorr. The driving force for sublimation, as is well-known, is the differential pressure between the ice interface and the chamber as seen in Eq. 1, where dm/dt is the sublimation rate, *A*_p_ is the cross-sectional area of the product, *P*_i_ is the pressure at the sublimating ice interface, *P*_c_ is the chamber pressure, and *R*_p_ is the mass transfer resistance.^22^

1 $$ \frac{\mathrm{dm}}{\mathrm{dt}}=\frac{A_{\mathrm{p}}\left({P}_{\mathrm{i}}-{P}_{\mathrm{c}}\right)}{R_{\mathrm{p}}} $$

The chamber pressure in the MVD and lyophilizer was 60 mTorr and 40 mTorr, respectively. Therefore, the corresponding pressure differential in the microwave drying process was higher (127 mTorr *vs*. 95 mTorr). However, using first-order modeling, this increase would only be enough to represent a 33.7% increase in driving force for MVD assuming the mass transfer resistance and vial area are held constant. Based on thermocouple/RTD (resistance temperature detector) performance, the primary drying time (primary drying only, see Fig. 5a and b) for MVD was reduced by approx. 83.3% (from 18 h in the lyophilizer to 3 h in the microwave vacuum dryer) suggesting that *T*_p_ differences may not be enough to explain the observed rapid dehydration.

Figure 5c shows the specific surface area and residual moisture comparison of lyophilized and microwave-dried cakes. Differences in specific surface area and/or moisture can inform whether there would be differences in the mass transfer resistance term. Cake collapse, which would reduce the mass transfer term, would also reduce the specific surface area and increase residual moisture. The center vials from the microwave drier had an SSA of 1.69 m^2^/g and the edge vials 1.35 m^2^/g. The center vials from the lyophilizer had an SSA of 1.49 m^2^/g and the edge vials 1.73 m^2^/g, demonstrating comparability between microwave-dried and freeze-dried samples. Based on first-order modeling, this would support that both sample sets had similar *R*_p_. Microwave-dried samples did have a slightly higher moisture (Fig. 5d) but did not increase so much as to suggest significant micro-collapse. Since the vial and fill volumes were held constant in both experiments, no difference in *A*_p_ is expected. Thus, some other factors may be influencing the overall reduction in cycle time, demonstrating the complexity of microwave heating. Several formulation parameters such as dielectric properties, microwave absorption, and depth of penetration may be crucial to understand the interaction between microwave and sample and were evaluated further.

### Rapid Sublimation Rate Due to Dielectric and Loss Factor

Our results demonstrated an increased sublimation rate in MVD, but at similar pressure differentials and mass transfer resistance as that of traditional freeze drying. Thus, to further understand drying mechanism, we also examined the microwave absorption models. As per Eq. 2, the power dissipated (*P*_d_) from microwave absorption is in terms of watts/m^3^ and is proportional to a constant *K*, the dielectric constant *ɛ*, the loss factor tan(*δ*), the microwave frequency *f*, and the electric field *E* (23,24).

2 $$ {P}_{\mathrm{d}}=K\ \varepsilon\ \tan \kern0.1em \delta\ f\ {E}^2 $$

Thus, we hypothesized that microwave energy being absorbed volumetrically could be part of the reason for faster drying. By volumetrically, we mean that microwave energy penetrates the entire three-dimensional structure of the product and is absorbed throughout the volume. Thus, larger-volume samples would absorb proportionally more microwave energy. This contrasts with conduction-based lyophilization where heat is transferred per unit area, and not per unit volume. For lyophilization, increasing the fill volume does not increase the heat transfer. Running a series of microwave cycles at increasing wattages, each containing a series of formulations with increasing sucrose content, we were able to measure the amount of water removed gravimetrically. Mass removed was then converted to wattage absorbed by multiplying by the enthalpy of sublimation (2840 J/g) and normalized by vial surface area as per Eq. 3**.**

3 $$ \frac{\mathrm{dq}}{\mathrm{dt}}=\Delta  {H}_s\left(\frac{\mathrm{dm}}{\mathrm{dt}}\right) $$

**Fig. 6:**
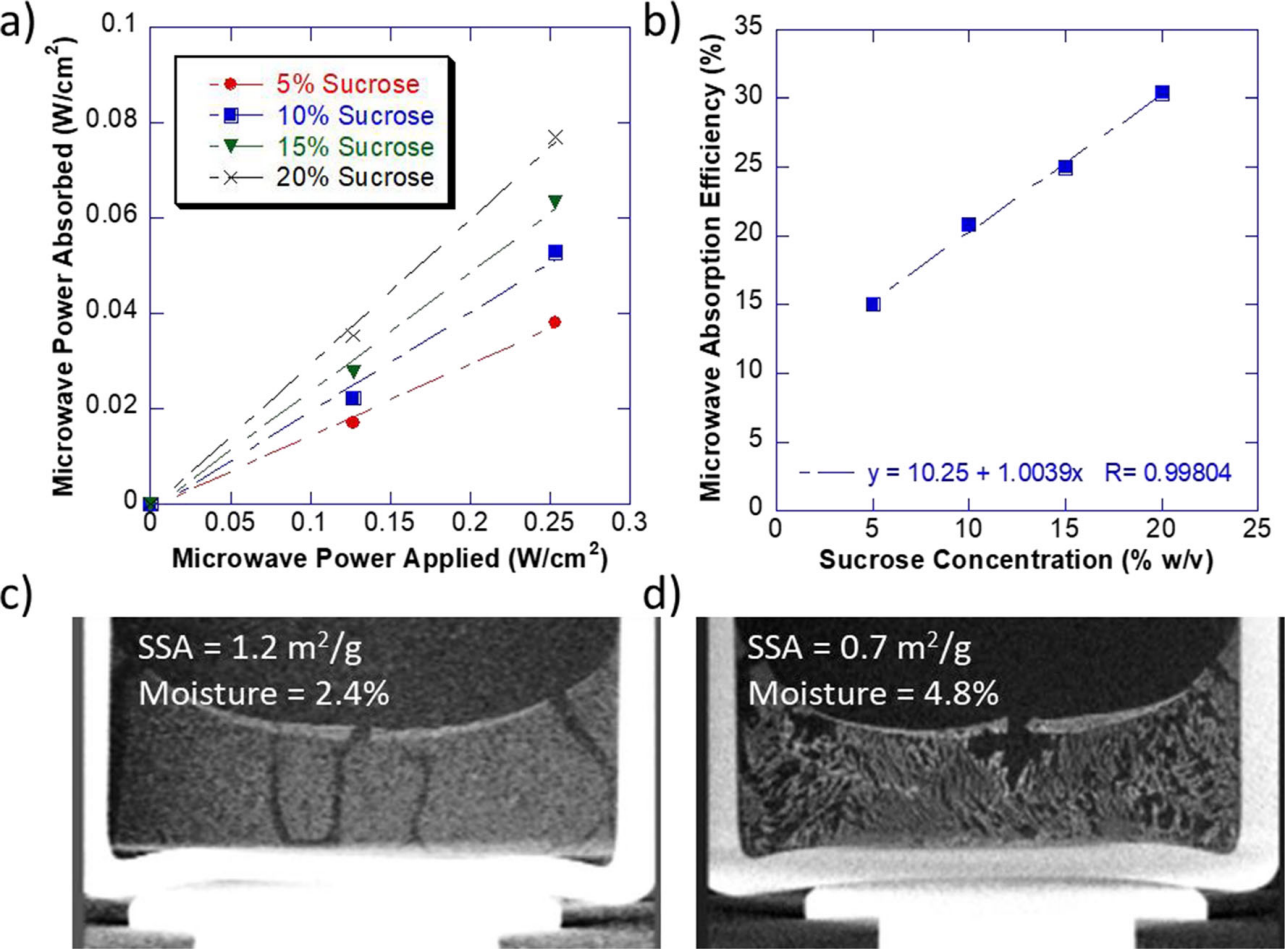
Heat transfer as a function of microwave power applied, dielectric, and loss factors. **a** The normalized microwave power absorbed as a function of normalized microwave power applied on a per area basis for various sucrose concentrations. **b** The microwave absorption efficiency, which represents the dielectric and/or loss factor, increases as sucrose concentration increases. MicroCT, SSA, and moisture for **c** lyophilized HDF and **d** microwave-dried HDF

The effects of radiation and other extraneous heat sources were determined in front runs where microwave energy was not applied. This background effect was then subtracted from each run with microwave to ensure that we only considered the energy provided by microwave interactions. Lastly, we normalized the microwave applied by the shelf area in the microwave dryer. The normalized microwave power absorbed was plotted against the normalized power applied for each of the different sucrose formulations (Fig. 6a). It was observed that the amount of microwave absorbed increased proportionally to the power applied, predicted by the electric field term in Eq. 2.

To further analyze the absorption efficiency, the slope of the curve in Fig. 6a, which is a ratio of the microwave absorbed to the microwave applied, was plotted as a function of sucrose concentration (Fig. 6b). Absorption efficiency increased linearly with increasing sucrose concentration. This is in line with expectation and correlates with formulation characteristics containing the dielectric and loss factor as defined in Eq. 2. The other relevant term for microwave frequency was held constant at 2.45 GHz in our case.

To further understand factors critical for faster drying under higher *R*_p_ values MVD was performed at higher wattage for a high disaccharide formulation (HDF, approx. 25% sugar composition). HDF was utilized specifically to evaluate the balance between the increase in microwave absorption with the counter increase in mass transfer resistance. Despite high product resistance associated with HDF, microwave drying time for HDF was similar to that achieved with 5% w/v sucrose. However, this time, we observed a difference in the cake structure, a reduction in specific surface area, and an increase in residual moisture, all suggesting some degree of micro-collapse (Fig. 6c and d) (25). In this case, product temperature for the microwave-dried HDF (− 29 °C) was warmer than the glass transition temperature (− 33 °C), suggesting that volumetric heating was accompanied by viscous drying where micro-collapses aid in reducing the mass transfer resistance. Thus, our data, illustrates that conductive heat transfer in freeze-drying can be overcome through radiative heat transfer. The interaction of microwave radiation with excipients serves as a key drying mechanism that has the potential to influence heat as well as mass transfer phenomenon thereby allowing faster drying.

### Compatibility of Microwave Vacuum Drying with Live Virus Product

**Fig. 7:**
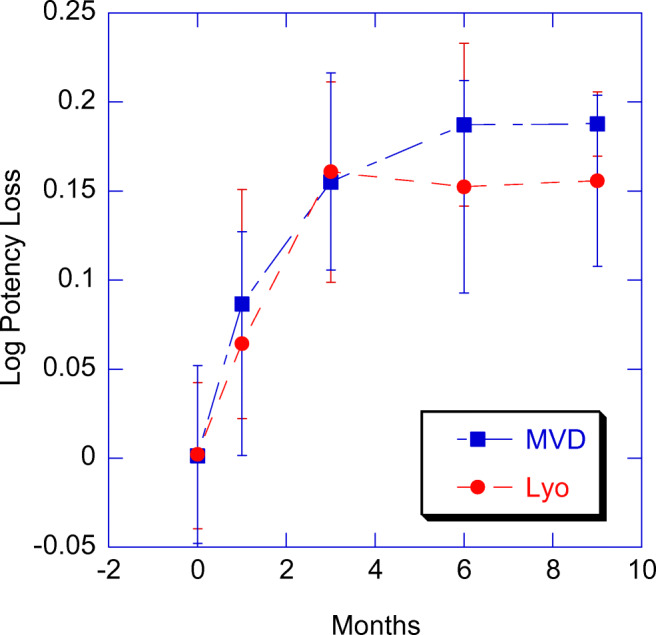
LVV stability profile for MVD and lyophilization. Live virus vaccine dried in the microwave vacuum dryer and lyophilizer were stored at 2–8 °C for up to 9 months. Log potency loss was compared to the time-zero relative potency result. Each error bar represents the standard deviation from *n* = 9 vials

To better understand the real-time impact of MVD on product stability, a live virus vaccine (LVV) was also evaluated for its potency as a function of long-term storage conditions post-drying. The chosen virus is known to be relatively unstable and sensitive to various process parameters. However, when dried either in the benchmark lyophilization cycle or in the microwave vacuum dryer, the potency loss at accelerated conditions was nearly identical over the course of 9 months (Fig. 7). Thus, for this virus under these specific conditions, microwave vacuum drying offers the potential for much faster drying compared to lyophilization, without impact to a key product quality attribute.

## DISCUSSION

The key driver behind drying has not changed since its first application for preserving food (26) and now lyophilization serves as the standard approach for stabilization of vaccines and biological medicinal products. Vial lyophilization, in general, has advanced to a more defined and characterized process from both a scientific as well as operational perspective. There are numerous procurement options for obtaining reliable, GMP manufacturing lines with automated vial filling and lyophilization equipment. Microwave heating, in contrast, is a relatively newer approach that was discovered during World War II and enjoyed a revival in 1980 for drying of potent, granular tablets and pharmaceuticals (27,28), and in recent years has been explored for preservation of probiotics and enzymes (23). The use of microwave-assisted drying for vaccines and biologics, however, is severely limited. The aim of this article is to share findings from our initial feasibility assessment of drying proteins and vaccines using microwave vacuum drying and to compare the MVD approach to conventional freeze-drying process using the first-principle model.

A systematic feasibility assessment demonstrated the potential for utilizing MVD as a faster alternative to freeze drying wherein the drying occurs in hours and not days. Gravimetric analysis demonstrated comparable sublimation pathways between MVD and lyophilization under proportionately partial drying conditions even though drying in MVD was approximately 87% faster than lyophilization (Fig. 2). No visual signs of meltback or dye diffusion were observed, and the cake visualization studies using phenol red demonstrated that this reduction in cycle time was achieved while the product remained properly frozen (Fig. 3a). Furthermore, using hemoglobin and catalase as surrogate proteins, post-drying appearance and protein activity was observed to be comparable between freeze-dried and microwave-dried samples (Fig. 3b and Fig. 4). These studies also illustrated the complexity of microwave heating and underscored the importance of several parameters of formulations such as penetration depth, dielectric, loss factor, and microwave absorption that are not relevant during standard freeze-drying process.

It has been well-known for several decades that the heat required for sublimation is the summation of conductive, convective, and radiant heat transfer mechanisms. Pikal *et al*. simplified these complex heat transfer equations by lumping the heat transfer sources into a single heat transfer coefficient *K*_v_ as seen in Eqs. 4 and 5:

4 $$ Q={K}_v{A}_v\left({T}_s-{T}_b\right) $$

5 $$ {K}_v={K}_c+{K}_r+{K}_g $$

where *Q* is the heat flow into the vial, *A*_v_ is the cross-sectional area of the vial, *T*_s_ is the shelf temperature, *T*_b_ is the product temperature, *K*_v_ is the vial heat transfer coefficient, *K*_c_ the contribution from conduction, *K*_r_ the contribution from radiation, and *K*_g_ the contribution from gas conduction (29). The authors subsequently evaluated these equations to summarize heat transfer in a traditional laboratory lyophilizer as shown in Eq. 6:

6 $$ Q={A}_v\left({K}_c+{1.10}^{-4}{e}_{\mathrm{s}}+{K}_g\right)\left({T}_s-{T}_b\right)+{A}_v{e}_{\mathrm{v}}\sigma \left({Ti}^4-{T}^4\right) $$

where *e*_s_ is the shelf surface emissivity, *e*_v_ is the vial top emissivity, *σ* is the Stefan-Boltzmann constant, *Ti*_l_ is the lid temperature or temperature of the above shelf, and *T* is the product temperature (29). Here, in this paper, we have demonstrated the use of microwave energy as another source of heat to drive sublimation in the freeze-drying process. Therefore, the heat flux equation can be re-written to add the microwave contribution as shown in Eq. 7:

7 $$ {Q}_{\mathrm{total}}={Q}_{\mathrm{conduction}}+{Q}_{\mathrm{convection}}+{Q}_{\mathrm{radiation}}+{Q}_{\mathrm{microwave}} $$

Using the power dissipated in Eq. 2 for *Q*_microwave_, and following the logic of Eqs. 4, 5, and 6, we can then define the heat transfer for microwave vacuum drying using Eq. 8**:**

8 $$ Q={A}_v\left({K}_c+{1.10}^{-4}{e}_s+{K}_g\right)\left({T}_s-{T}_b\right)+{A}_v{e}_v\sigma \left({Ti}^4-{T}^4\right)+K\ \varepsilon\ \tan \kern0.1em \delta\ f\ {E}^2 $$

Understanding how these newly introduced parameters are impacted by equipment design and excipient selection are critical for optimizing microwave vacuum drying for pharmaceutical products as they determine the predominant heat transfer mechanism be it radiation or conduction or a combination thereof, while maintaining critical quality attributes. Penetration depth, for example, is crucial for uniform drying and is dependent on the proper selection of microwave wavelength. Penetration depth is determined by material properties and directly proportional to microwave wavelength (12.2 cm at 2.45 GHz) (30). Similarly, dielectric properties (dielectric constant *ε*′ and loss factor *ε*″) are dependent on the composition of sample and allows quantification of interaction at a molecular level wherein *ε*′ is indicative of capacitive energy stored while ε″ demonstrates potential for this energy conversion to heat in response to microwave exposure, respectively (23,31). Various factors affect *ε*′ and ε″, including, but not limited to, temperature, water content, solute percentage, and phase of material. At higher moisture content and at temperatures above the freezing point, for example, heat transfer is expected to be higher than under the dry state. Thus, in contrast to freeze-drying, a change in moisture and temperature as the drying progresses results in corresponding change in heat transfer by virtue of changing dielectric properties. For example, as moisture is reduced in the drying process, it is expected that microwave absorption would also decrease in what could be hypothesized as a self-regulation by microwave. In addition, it should be noted that dielectric properties of formulation excipients are non-additive (31). Thus, to better understand the mechanism of interaction, a series of increasing sucrose formulations were dried in microwave vacuum dryer and characterized (Fig. 5 and Fig. 6). When empirical data was analyzed against the first-principle heat and mass transfer model used to explain freeze-drying phenomenon as well as radiative drying process (Eqs. 1, 2, 4 and 7), we hypothesize that rapid drying in MVD is attributed to effective energy transfers in a volumetric format with potential for viscous drying that is not constrained by the apparent heat coefficient of a vial, *K*_v_. Furthermore, the presence of higher solute concentration in MVD results in greater microwave absorption efficiency. This contrasts with freeze-drying phenomenon wherein the heat transfer for sublimation occurs through conduction from the bottom of a vial as an intrinsic property of the vial and increasing solute concentration further slows down the sublimation due to an increase in mass transfer resistance.

Given the intrinsic instability of live virus vaccines, a comparison with lyophilization was deemed crucial to comprehend the potential for thermal runaway (*i.e.* hot spots) and its corresponding impact on long-term shelf life of the vaccine. As mentioned above and in contrast to the freeze-drying process where primary drying typically occurs at product temperature at or below its glass transition temperature, MVD may occur at or above its glass transition temperature with a self-modulated heat transfer that changes as dielectric properties of formulation changes with drying process advancement. The potential for product degradation prompted us to perform the stability studies and under the given experimental conditions, no differences were observed between microwave- and lyo-dried LVV over the course of 9-month storage conditions (Fig. 7).

The current SARS-CoV2 pandemic, evolving pipeline, need for specialty medicine targeting high unmet medical needs and other factors, highlights the ever-increasing value of on-demand manufacturing. Furthermore, the SARS-CoV2 pandemic, which will demand unprecedented number of manufactured doses, demonstrates the critical need to reduce cycle time and increase throughput capacity in high-volume responsive scenarios. As a pharmaceutical community and freeze-drying scientists, it is imperative for us to explore and adopt faster drying alternatives while challenging traditional drug product manufacturing approaches. This report forms the very first example in the literature on the use of MVD for stabilizing LVVs (live virus vaccines). The article also provides a scientific foundation and identifies some of the critical issues related to the drying process pertaining to the development of stable pharmaceutical formulations. Even though MVD is a relatively new drying approach compared to freeze drying, the technology has attracted enough attention to warrant further exploration as various questions are still unanswered. Findings from our study showcase the compatibility of MVD process for drying sensitive biologics and vaccines, and radiative heat transfer may serve as a faster and potentially a more energy-efficient alterative to conductive drying. The reader is also reminded that microwave radiation does not possess any intrinsic heat and any dielectric heating is a function of its interaction with formulation components as illustrated in this study (32).

The MVD units are currently made to address food safety concerns and are not designed to be used in a more stringently controlled GMP environment for drying pharmaceutical products, and a lab-scale prototype was utilized for this study. Furthermore, the unit evaluated in this publication was specifically designed as a fit-for-purpose prototype and as such was limited to drying approximately 300 vials or less. Lab-scale lyophilizers, on the other hand, are built to dry approximately 1000–2000 vials per cabinet. Therefore, in addition to overcoming GMP barriers, further work is needed to demonstrate scalability to more commercially applicable batch sizes. Otherwise, the reduction in cycle time would be offset by the number of vials that can be processed at once. Still, due to the potential for semi-continuous manufacturing, we do not expect the commercial microwave dryers to need to be as large as traditional commercial lyophilizers.

Other challenges that need to be overcome with the scale-up and eventual GMP implementation of MVD include in-chamber stoppering and the inclusion of a freezing step. For this prototype, the chamber vacuum was released to the atmosphere and vials quickly stoppered by hand. This has the obvious potential to increase residual moisture as a result of the prototype design and not the drying process. However, this was deemed acceptable for our initial exploration as it would be considered worst-case. Secondly, traditional lyophilization cabinets include the ability to freeze on the shelves. This cannot occur within our prototype because metal shelves would negatively react with the microwave field. Both the stoppering and freezing challenges will need to be resolved to successfully scale to GMP equipment.

On-demand manufacturing of biologics and vaccines require the need for exploring and alternate drying technologies to standard lyophilization (33), and MVD represents a viable alternative. Furthermore, our findings demonstrated the potential benefits and challenges of MVD relative to conventional vial-based lyophilization that may eventually enable semi-continuous and on-demand manufacturing by virtue of its faster drying time.

## CONCLUSIONS

Merck & Co., Inc. (West Point, PA, USA) has explored the compatibility of the microwave vacuum drying process with two different proteins and a live virus vaccine and observed that the process has the potential to be broadly applicable to the same product types that today rely on vial lyophilization. Using a lab-scale unit, our results demonstrate that MVD serves as one of the most rapid dehydration approaches that can reduce freeze-drying cycle time by ≥ 80%. Product critical quality attributes (CQAs) were found to be comparable between a lyo-dried and MVD sample for all cases studied in this report. This contrasts with standard vial freeze drying that is a slow batch drying process requiring a cycle time in the order of days, often requiring scale-up (*i.e.*, capital) to multiple units in a given manufacturing facility and thus prompting the need for faster alternatives to pharmaceutical freeze drying. By virtue of its rapid drying, MVD has the potential to enable semi-continuous manufacturing, and our hope is that this work serves as a foundational prompt to develop a more optimized drying technology that enables flexible manufacturing. The challenge ahead is to build upon the fundamental heat and transfer principles associated with MVD and lyophilization to eventually couple more effective heat transfer mechanism associated with radiative drying process with the optimal process controls obtained in lyophilization while maintaining compliance with the cGMP regulations. Such flexible rapid cGMP drying, once established, would not only help reduce the overall operational costs but would also better serve our patient needs through on-demand manufacturing.

## References

[1] Kasper JC, Winter G, Friess W (2013). Recent advances and further challenges in lyophilization. Eur J Pharm Biopharm.

[2] Gieseler H, Kessler WJ, Finson M, Davis SJ, Mulhall PA, Bons V, Debo DJ, Pikal MJ (2007). Evaluation of tunable diode laser absorption spectroscopy for in-process water vapor mass flux measurements during freeze drying. J Pharm Sci.

[3] Geidobler R, Winter G (2013). Controlled ice nucleation in the field of freeze-drying: fundamentals and technology review. Eur J Pharm Biopharm.

[4] Goldman JM, Chen X, Register JT, Nesarikar V, Iyer L, Wu Y, Mugheirbi N, Rowe J (2019). Representative scale-down lyophilization cycle development using a seven-vial freeze-dryer (MicroFD®). J Pharm Sci.

[5] Bhambhani A, Blue JT. Lyophilization strategies for development of a high-concentration monoclonal antibody formulation: benefits and pitfalls, Am. Pharm. Rev. 2010; Feb: 31–38.

[6] Kapoor Y, Meyer R, Meyer B, DiNunzio JC, Bhambhani A, Stanbro J, et. al. Flexible manufacturing: the future state of drug product development and commercialization in the pharmaceutical industry. J. Pharm Innovation; 2020. 10.1007/s12247-019-09426-z.

[7] Lovalenti PM, Anderl J, Yee L, Nguyen V, Ghavami B, Ohtake S, Saxena A, Voss T, Truong-Le V (2016). Stabilization of live attenuated influenza vaccines by freeze drying, spray drying, and foam drying. Pharm Res.

[8] Walters RH, Bhatnagar B, Tchessalov S, Izutsu KI, Tsumoto K, Ohtake S (2014). Next generation drying technologies for pharmaceutical applications. J Pharm Sci.

[9] Van Bockstal PJ, De Meyer L, Corver J, Vervaet C, De Beer T (2017). Noncontact infrared-mediated heat transfer during continuous freeze-drying of unit doses. J Pharm Sci.

[10] De Meyer L, Van Bockstal PJ, Corver J, Vervaet C, Remon JP, De Beer T (2015). Evaluation of spin freezing versus conventional freezing as part of a continuous pharmaceutical freeze-drying concept for unit doses. Int J Pharm.

[11] Wanning S, Süverkrüp R, Lamprecht A (2015). Pharmaceutical spray freeze drying. Int J Pharm.

[12] Freeze drying of microspheres by spray freezing and dynamic bulk freeze drying. http://meridion-technologies.de/bilder/pdf/Folder_LAB_EN.pdf. Accessed 23 Nov 2020.

[13] LYNFINITY Continuous Aseptic Spray-Freeze-Drying. https://ima.it/pharma/machine/lynfinity/ Accessed 23 Nov 2020.

[14] Sebastião IB, Bhatnagar B, Tchessalov S, Ohtake S, Plitzko M, Luy B, Alexeenko A (2019). Bulk dynamic spray freeze-drying part 1: modeling of droplet cooling and phase change. J Pharm Sci.

[15] Gitter JH, Geidobler R, Presser I, Winter G (2018). Significant drying time reduction using microwave-assisted freeze-drying for a monoclonal antibody. J Pharm Sci.

[16] Gitter JH, Geidobler R, Presser I, Winter G (2019). Microwave-assisted freeze-drying of monoclonal antibodies: product quality aspects and storage stability. Pharmaceutics..

[17] Lale SV, Goyal M, Bansal AK (2011). Development of lyophilization cycle and effect of excipients on the stability of catalase during lyophilization. Int J Pharm Investig.

[18] Luthra S, Obert JP, Kalonia DS, Pikal MJ (2007). Investigation of drying stresses on proteins during lyophilization: differentiation between primary and secondary-drying stresses on lactate dehydrogenase using a humidity controlled mini freeze-dryer. J Pharm Sci.

[19] Bhambhani A, Kumar CV (2006). Tuning the properties of Hb intercalated in the galleries of α-ZrP with ionic strength: improved structure retention and enhanced activity. Chem Mater.

[20] Zhou D, Shang S, Tharp T, Jameel F, Sinha K, Nere NK (2019). Leveraging lyophilization modeling for reliable development, scale-up and technology transfer. AAPS PharmSciTech.

[21] Pikal MJ, Pande P, Bogner R, Sane P, Mudhivarthi V, Sharma P (2018). Impact of natural variations in freeze-drying parameters on product temperature history: application of quasi steady-state heat and mass transfer and simple statistics. AAPS PharmSciTech.

[22] Tang X, Pikal MJ (2004). Design of freeze-drying processes for pharmaceuticals: practical advice. Pharm Res.

[23] Durance T, Yaghmaee P. Comprehensive biotechnology 2nd Ed. 2011; Vol 4: 617–628.

[24] Nair BKS, Parkes GMB, Barnes PA, Sibley MJN, Bond G (2006). Development of a novel instrument for microwave dielectric thermal analysis. Review of Scientific Instruments.

[25] Nail SL, Jiang S, Chongprasert S, Knopp SA (2002). Fundamentals of freeze-drying. Pharm Biotechnol.

[26] Hayashi H (1989). Drying technologies of foods: their history and future. Dry Technol.

[27] Cliff M. Use of microwaves to dry pharmaceutical granules. Proceedings of IEE Colloqium -Electricity in Pharmaceuticals and Specialised Organics, 1986; pp. 1–4.

[28] McLoughlin C, McMinn W, Magee T (2003). Microwave drying of multi-component powder systems. Dry Technol.

[29] Pikal MJ, Roy ML, Shah S (1984). Mass and heat transfer in vial freeze-drying of pharmaceuticals: role of the vial. J Pharm Sci.

[30] Venkatesh MS, Raghavan GSV (2004). An overview of microwave processing and dielectric properties of agri-food materials. Biosyst Eng.

[31] Yaghmaee P, Durance T (2002). Predictive equations for dielectric properties of NaCl, D-sorbitol and sucrose solutions and surimi at 2450 MHz. J Food Sci.

[32] Durance T, Noorbakhsh R, Sandberg G., Saenz-Garzam N. Microwave drying of pharmaceuticals, In Drying technologies for biotechnologies and pharmaceutical applications. Ohtake, S; Izutsu, Ken-ichi; Lechuga-Ballesteros, D. 2020; pp. 239.

[33] Bhambhani A, Antochshuk V Vaccines and microorganisms. In Drying technologies for biotechnologies and pharmaceutical applications. Ohtake, S; Izutsu, Ken-ichi; Lechuga-Ballesteros, D. 2020; pp. 121–134.

